# Improved Life Preserver

**Published:** 1845-12

**Authors:** 


*Improved Life Preserver.*—We have lately examined a newly invented Life Preserver, called the *Nautilus*, which appears to us so much superior to any hitherto proposed, and so perfect, that we cannot refrain from commending it to our readers, and, through them, to their friends and the Western public generally; who from the vast extent and multiplied dangers of our navigable rivers, are deeply interested. It consists of a gum elastic tube several inches in diameter, and long enough, when stretched out, to surround the chest of a man, while, by pressing its ends towards each other, with its aperture open, it is so reduced in length, in diameter remaining the same, that it may be carried in the coat pocket. Within it there are two coiled wires, similar to that within the cushion of a sofa, which, by drawing the ends from each other, have their coils separated, so as to give the length just mentioned, while the diameter of the tube remains nearly unaltered. Of course atmospheric air flows in through the hole at one end, to which there is a plug or stopper, not to keep the air in but the water out; for as long as that is done, and the tube is kept stretched round the body it necessarily retains its air, and consequently its buoyancy. Should it be punctured, unless the holes be large enough to let water pass in, no harm will be done, for the wires will keep the sides from collapsing. In fact, nothing could be more simple and beautiful than the principle on which it acts; and no one can examine it without feeling confidence in its preserving power. We are not surprised, then, to find it strongly recommended by the American Shipwreck Society, and the American Institute. We hope to see it generally adopted on the lakes and rivers of the interior.—*Western Journal.*

